# Trajectories and changes in individual items of positive and negative syndrome scale among schizophrenia patients prior to impending relapse

**DOI:** 10.1038/s41537-018-0056-6

**Published:** 2018-06-20

**Authors:** Dai Wang, Srihari Gopal, Susan Baker, Vaibhav A. Narayan

**Affiliations:** 1 R&D Information Technology, Janssen Research & Development, LLC, Titusville, NJ USA; 2 Neuroscience Therapeutic Area, Janssen Research & Development, LLC, Titusville, NJ USA

## Abstract

Effective early detection of impending relapse may offer opportunities for early interventions to prevent full relapse in schizophrenia patients. Previously reported early warning signs were not consistently validated by prospective studies. It remains unclear which symptoms are most predictive of relapse. To prioritize the symptoms to be captured by periodic self-report in technology-enabled remote assessment solutions for monitoring symptoms and detecting relapse early, we analyzed data from three relapse-prevention studies to identify individual items of the Positive and Negative Syndrome Scale (PANSS) that changed the most prior to relapse and to understand exactly when these symptoms manifested. Relapse was defined by a composite endpoint: hospitalization, suicidal/homicidal ideation, violent behavior, a 25% increase in the PANSS total score, or a significant increase in at least one of several pre-specified PANSS items. Longitudinal mixed effect models were applied to model the trajectories of individual PANSS items before relapse. Among 267 relapsed patients, the PANSS items that increased the most at relapse from randomization did not differ much by different relapse reasons or medications. A subset of seven PANSS items, including delusions, suspiciousness, hallucinations, anxiety, excitement, tension, and conceptual disorganization, had on average > 1-point of increase at relapse. The trajectories of these items suggested these items started to increase 7–10 days before relapse and reached on average 1-point of increase 0.3 ~ 1.2 days before relapse. Our results indicated that a subset of PANSS items could be leveraged to develop remote assessment solutions for monitoring symptoms and detecting relapse early in schizophrenia patients.

## Symptom monitoring: spotting the signs of relapse

Identifying the symptoms that are most predictive of relapse in patients with schizophrenia could guide novel early intervention strategies. High relapse rates, particularly during the first 5 years after illness onset, can lead to treatment resistance as well as functional impairment and decreased quality of life. The Positive and Negative Syndrome Scale (PANSS) is widely used to assess the severity of schizophrenia symptoms but it is too long to be used frequently for symptom monitoring. Dai Wang and colleagues at Janssen Research & Development, New Jersey, USA, analyzed data from three studies involving 267 relapsed patients. They found increases in the scores of seven PANSS items between 7 and 10 days before a relapse. Assessing these items weekly, by using mobile communication or wearable devices, could improve the accuracy of relapse prediction and provide opportunities to prevent it.

## Introduction

The disease course of schizophrenia is often characterized by frequent relapse of psychotic symptoms for the majority of patients. Repeated relapses may lead to treatment resistance, cognitive and functional impairment, decreased quality of life, and increased economic burden.^1–8^ Suicide rate is high in schizophrenia with 4.9% of patients taking their own lives eventually.^9^ Because of these risks, relapse prevention is a key component of schizophrenia patient management. Effective early detection of relapse through symptom monitoring may offer the opportunity of early interventions to prevent full relapse.^10–14^

There has been a growing interest in using mobile communication devices (such as smartphones and tablets) as well as wearable devices (such as actigraphs and smart watches) to monitor mental health and deliver mental health care.^15–19^ Researchers have started to explore the feasibility of using mobile devices to monitor the symptoms of severe mental illnesses such as schizophrenia, schizoaffective disorder, and bipolar disorder.^20–24^ A pilot study^23^ used an Android smartphone application called ClinTouch to collected self-reports on 12 items of the Positive and Negative Syndrome Scale (PANSS)^25^ and two items of the Calgary Depression Scale six times daily for a week in 44 participants (36 with schizophrenia). The overall compliance was good with 82% of the participants completed at least 33% of all possible data-points. Several items, including delusions, hallucinations, suspiciousness, anxiety, and hopelessness, were correlated with corresponding items from interview rating scales, supporting that smartphone applications can be used to collect clinically meaningful self-assessment of psychiatric symptoms.

Mobile devices can collect continuous passive sensing^16^ and periodic self-reports data. CrossCheck^24^ is the first smartphone-sensing system that uses continuous passive sensing and periodic self-reports to monitor and assess mental health changes in schizophrenia. Preliminary data of CrossCheck have indicated statistically significant associations between automatically tracked behavioral features related to sleep, mobility, conversations, smartphone usage, and self-reported indicators of mental health in schizophrenia. Although it may be attractive to use passive sensing data to predict relapse in schizophrenia, combining this data with periodic self-reports on symptoms of schizophrenia may improve the accuracy of relapse prediction. However, owing to fatigue factor and cognitive impairment inherent in the disease process, having patients to provide frequent and lengthy self-reports on all symptoms of schizophrenia would be too much a burden and thus infeasible. We need to focus on the symptoms that are most predictive of impending relapse in schizophrenia.

Early warning signs of relapse in schizophrenia have been identified through both prospective and retrospective studies.^26–28^ These early warning signs included both psychotic and non-psychotic symptoms. In a prospective study^27^ where clinicians recorded whether the early signs exist during weekly treatment visits prior to decompensation in 47 schizophrenic patients, the most commonly observed symptoms included hallucinations (53%), suspiciousness (43%), change in sleep (43%), anxiety (38%), cognitive inefficiency (26%), anger/hostility (23%), somatic symptoms or delusions (21%), thought disorder (17%), disruptive inappropriate behavior (17%), and depression (17%). The majority of these symptoms were psychotic symptoms. This is different from the early warning signs identified through retrospective studies where non-psychotic symptoms, such as having trouble sleeping, having trouble concentrating, loss of appetite, and feeling depressed, ranked higher.^26,29,30^ A few prospective studies have been performed to assess the predictive validity of the early warning signs but yielded mixed results.^28,31–41^ Although the inconsistent results may be due to the actual symptoms monitored and the frequency of the monitoring,^42^ it remains unclear which symptoms are best predictive of impending relapse in schizophrenia.

Current standard care for schizophrenia is based on brief assessments of the patients’ clinical symptoms during outpatient visits. Comprehensive scales for assessing disease severity of schizophrenia, such as the PANSS, assess the full spectrum of schizophrenia symptoms but are time consuming, which prevents frequent administration. More often in a typical psychiatric practice, patients’ disease severity is tracked by a global assessment of function score, which only measures the overall disease severity without assessing the individual symptoms of schizophrenia systematically. This is insufficient for tracking the individual symptoms and understanding which symptoms changed the most immediately before relapse.

Data presented in prior studies indicate that onset of relapse is abrupt in context of the frequency at which patients are seen at the clinic.^4^ Under standard care patients with stable schizophrenia may be seen monthly or even less frequently, which is not sufficient to capture the early signs of relapse. The frequency of clinic visits in most parts of the world with limited availability of psychiatrists is even lower. When a patient presents to the hospital owing to relapse, it may have been weeks or even months since the patient’s last pre-relapse visit. Although psychiatric symptoms may have changed during this period, they often go undetected. Understanding the time course of symptom worsening before a relapse can help to determine how frequently a patient’s symptoms should be monitored.

In this study, we analyzed data from three relapse-prevention studies conducted by Janssen to identify individual PANSS items that changed the most prior to relapse. We also model the trajectories of individual PANSS items using both linear and non-linear mixed effect models to understand exactly when these symptoms manifested. Our primary objective was to determine: (1) which individual symptoms from the PANSS could be used to predict an impending relapse and (2) the time course of changes in these symptoms prior to relapse. The results from these analyses provide basis for developing technology-enabled remote assessment solutions for monitoring symptoms and detecting relapse early in schizophrenia patients.

## Results

### Demographics and characteristics of patients experienced a relapse during double-blind phase

Among 907 patients who were randomized and included in current analysis, a total of 267 patients experienced a relapse during the double-blind phase of the three studies. Among the 267 relapsed patients, 63 patients’ relapse was defined by a psychiatric event such as hospitalization, suicidal/homicidal ideation, or aggressive behavior, 65 patients met the criterion of a significant increase in at least one of several pre-specified PANSS items, and the remaining 139 patients met the criterion of a significant increase in the PANSS total score but did not meet the criterion on the pre-specified PANSS items. Compared with the patients who did not experience a relapse during the studies, no significant difference was observed in demographics and baseline characteristics except the relapsed patients had higher PANSS total scores at the baseline of the double-blind phase (Table 1).

**Table 1:** Demographics and baseline characteristics in patients who did and did not experience a relapse during the double-blind phase of the three studies

	Relapse^1^ (*N*=267)	Non-relapse (*N*=640)	*P* value^2^
Age, years	38.4 (±10.8)	38.5 (±11)	0.99
Male sex	158 (59.2%)	404 (63.1%)	0.26
Race			
White	178 (66.7%)	404 (63.1%)	n/a^3^
Black	41 (15.4%)	94 (14.7%)	
Asian	29 (10.9%)	56 (8.8%)	
Other	19 (7.1%)	86 (13.4%)	
Age at diagnosis, years	26.8 (±9.1)	27 (±8.9)	0.79
Body Mass Index, kg/m^2^	27.2 (±6.3)	26.4 (±5.4)	0.2
PANSS total score at double-blind phase baseline	54.9 (±10.7)	52.5 (±11.1)	**0.0021**

^1^Data shown are mean (±standard deviation) for continuous variables (age, age at diagnosis, body mass index, and PANSS total score at double-blind phase baseline) and *N* (%) for categorical variables (sex, race).

^2^*P* values are from two-sided Wilcoxon rank sum test for continuous variables (age, age at diagnosis, body mass index, and PANSS score at double-blind phase baseline) and *Χ*^2^ test for sex.

^3^*P* value for race is not calculated because patients in India were followed-up for a shorter period in study NCT00086320 owing to a late start of enrollment at sites in India.

### PANSS items with most increases at relapse

**Fig. 1:**
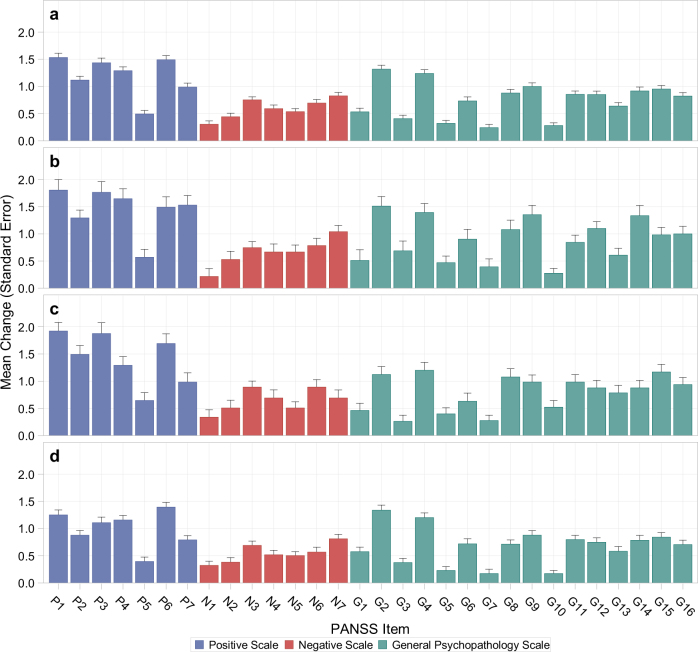
Increases in individual PANSS items at relapse from randomization in patients with different relapse reasons: **a** all relapsed patients (*N* = 255); **b** relapse defined by hospitalization, suicidal/homicidal ideation, or aggressive behavior (*N* = 51); **c** relapse defined by a significant increase in at least one of the pre-specified PANSS items (*N* = 65); **d** relapse defined by a significant increase in the PANSS total score but not in any of the pre-specified PANSS items (*N* = 139). The number of patients with a PANSS assessment at relapse was 255 instead of 267 because we were not able to obtain a PANSS assessment within 7 days of relapse for 12 out of the 63 patients whose relapse was defined by hospitalization, suicidal/homicidal ideation, or aggressive behavior. PANSS items: P1 – Delusions, P2 – Conceptual disorganization, P3 – Hallucinations, P4 – Excitement, P5 – Grandiosity, P6 – Suspiciousness, P7 – Hostility, N1 – Blunted affect, N2 – Emotional withdrawal, N3 – Poor rapport, N4 – Passive-apathetic social withdrawal, N5 – Difficulty in abstract thinking, N6 – Lack of spontaneity & flow of conversation, N7 – Stereotyped thinking, G1 – Somatic concern, G2 – Anxiety, G3 – Guilt feelings, G4 – Tension, G5 – Mannerisms & posturing, G6 – Depression, G7 – Motor retardation, G8 – Uncooperativeness, G9 – Unusual thought content, G10 – Disorientation, G11 – Poor attention, G12 – Lack of judgement & insight, G13 – Disturbance of volition, G14 – Poor impulse control, G15 – Preoccupation, G16 – Active social avoidance

Among the 267 relapsed patients, a subset of seven PANSS items had on average > 1-point of increase at relapse from randomization (Fig. 1a, Table 2). These seven PANSS items included P1 (delusions), P6 (suspiciousness), P3 (hallucinations), G2 (anxiety), P4 (excitement), G4 (tension), and P2 (conceptual disorganization). Similar patterns were observed in patients with different relapse reasons (Fig. 1b–d). At least six out of the top seven PANSS items that had the most increases at relapse in each of the three groups overlapped with the top seven items identified in all relapsed patients (Supplementary Table 2). Although more of the patients receiving placebo experienced a relapse than those receiving paliperidone, the same seven PANSS items had the most increases at relapse in both groups (Table 2). Six out of the top seven PANSS items that had the most increases at relapse in the patients receiving the oral extended-release (ER) formulation of paliperidone and in those receiving one of the two long-acting injectable formulations also overlapped with the top seven PANSS items identified in all relapsed patients (Supplementary Table 3). These results suggested the PANSS items that had the most increases at relapse did not differ much in patients having different relapse reasons or receiving different treatments.

**Table 2:** Increases in individual PANSS items at relapse from randomization in relapsed patients receiving paliperidone and those receiving placebo

PANSS item	All relapsed patients (*N*=255)		Patients receiving paliperidone (*N*=69)		Patients receiving placebo (*N*=186)	
	Rank	Mean change (standard error)	Rank	Mean change (standard error)	Rank	Mean change (standard error)
**P1 Delusions**	**1**	**1.53 (0.08)**	**1**	**1.35 (0.15)**	**1**	**1.60 (0.09)**
**P6 Suspiciousness**	**2**	**1.49 (0.08)**	**2**	**1.29 (0.15)**	**2**	**1.56 (0.09)**
**P3 Hallucinations**	**3**	**1.44 (0.09)**	**3**	**1.26 (0.18)**	**3**	**1.50 (0.10)**
**G2 Anxiety**	**4**	**1.32 (0.07)**	**4**	**1.26 (0.14)**	**5**	**1.34 (0.08)**
**P4 Excitement**	**5**	**1.29 (0.07)**	**6**	**1.07 (0.14)**	**4**	**1.37 (0.08)**
**G4 Tension**	**6**	**1.24 (0.07)**	**7**	**1.06 (0.13)**	**6**	**1.31 (0.08)**
**P2 Conceptual disorganization**	**7**	**1.12 (0.07)**	**5**	**1.09 (0.14)**	**7**	**1.13 (0.08)**
G9 Unusual thought content	8	1.00 (0.07)	18	0.71 (0.13)	8	1.11 (0.08)
P7 Hostility	9	0.99 (0.07)	16	0.72 (0.12)	9	1.09 (0.09)
G15 Preoccupation	10	0.95 (0.06)	8	0.94 (0.13)	10	0.96 (0.07)
G14 Poor impulse control	11	0.92 (0.07)	9	0.87 (0.15)	11	0.94 (0.08)
G8 Uncooperativeness	12	0.88 (0.07)	15	0.74 (0.14)	12	0.93 (0.08)
G11 Poor attention	13	0.85 (0.06)	20	0.65 (0.13)	13	0.93 (0.07)
G12 Lack of judgment & insight	14	0.85 (0.06)	19	0.71 (0.12)	14	0.90 (0.07)
N7 Stereotyped thinking	15	0.83 (0.06)	13	0.81 (0.13)	15	0.83 (0.07)
G16 Active social avoidance	16	0.82 (0.06)	12	0.84 (0.12)	16	0.82 (0.07)
N3 Poor rapport	17	0.75 (0.06)	10	0.86 (0.13)	17	0.72 (0.06)
G6 Depression	18	0.73 (0.07)	11	0.84 (0.15)	18	0.69 (0.08)
N6 Lack of spontaneity & flow of conversation	19	0.69 (0.06)	17	0.72 (0.12)	19	0.68 (0.08)
G13 Disturbance of volition	20	0.64 (0.06)	23	0.54 (0.12)	20	0.68 (0.07)
N4 Passive-apathetic social withdrawal	21	0.59 (0.07)	14	0.77 (0.13)	24	0.53 (0.07)
N5 Difficulty in abstract thinking	22	0.54 (0.05)	25	0.42 (0.12)	21	0.58 (0.06)
G1 Somatic concern	23	0.53 (0.07)	24	0.48 (0.13)	22	0.55 (0.08)
P5 Grandiosity	24	0.49 (0.06)	26	0.39 (0.12)	23	0.53 (0.08)
N2 Emotional withdrawal	25	0.44 (0.06)	21	0.62 (0.12)	27	0.38 (0.08)
G3 Guilt feelings	26	0.41 (0.06)	28	0.26 (0.10)	25	0.46 (0.08)
G5 Mannerisms & posturing	27	0.32 (0.05)	29	0.16 (0.10)	26	0.38 (0.06)
N1 Blunted affect	28	0.31 (0.06)	22	0.54 (0.11)	29	0.22 (0.07)
G10 Disorientation	29	0.28 (0.05)	30	0.13 (0.09)	28	0.34 (0.06)
G7 Motor retardation	30	0.24 (0.06)	27	0.30 (0.11)	30	0.22 (0.07)

Comparing the increases in individual PANSS items between from the last pre-relapse visit to relapse and from randomization to the last pre-relapse visit, most of the increases occurred after the last pre-relapse visit. Although the individual PANSS items had very little increases at the last pre-relapse visit, the same seven PANSS items had the most increases at this visit, suggesting that these seven items also started to increase earlier than other items prior to impending relapse (Supplementary Table 4).

### Trajectories of PANSS item increases before relapse

**Fig. 2:**
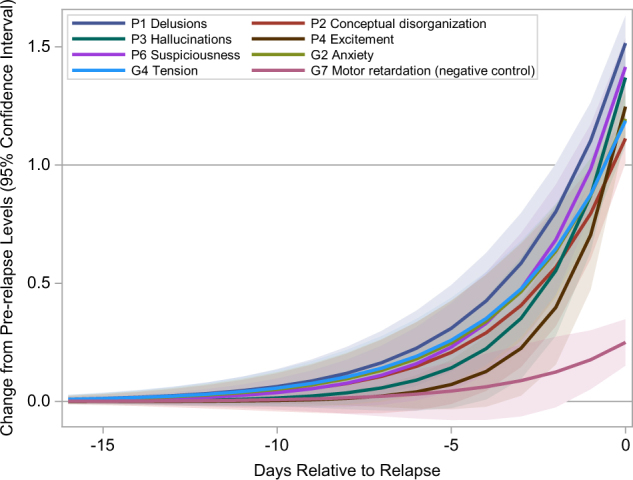
Trajectories of the changes from pre-relapse levels in the seven PANSS items that had the most increases at relapse. The trajectories were estimated from non-linear mixed effect models

To better understand when exactly these items started to increase before relapse, we modeled the trajectories of the individual PANSS items after realigning the patient observations by their time of observation as days relative to relapse. The trajectories of individual PANSS items before relapse estimated from the linear and non-linear mixed effect models were similar (Supplementary Figure 1). We focused on the trajectories estimated from the non-linear mixed effect models because the parameters of these models could be easily interpreted. Figure. 2 shows the trajectories of change from pre-relapse levels and their 95% confidence intervals of the seven PANSS items that changed most as well as a PANSS item with little increase (G7 (Motor retardation)) at relapse. The trajectories of the PANSS items that changed most at relapse suggested that these items started to increase ~7–10 days before relapse. The trajectories of these seven PANSS items were similar between patients receiving paliperidone and those receiving placebo (Supplementary Figure 2), suggesting the time courses of these individual PANSS items before relapse were similar in patients receiving different treatments.

The *b* parameter in the non-linear mixed effect models represented the number of days before relapse when the individual PANSS items had 1-point of increase from its pre-relapse level. The *b* parameter estimates of the seven PANSS items that had the most changes at relapse were < 0 (Table 3), indicating these PANSS items would have on average > 1-point of increase before relapse. The number of days before relapse when the individual PANSS item had on-average 1-point of increase from its pre-relapse level ranged from 0.33 to 1.17 for the seven PANSS items.

**Table 3:** The *b* parameter (time of one-point Increase as days relative to relapse) estimated from non-linear mixed effect models for individual PANSS items

Rank	PANSS item	Estimate of *b*	Standard error
**1**	**P1 Delusions**	**−1.17**	**0.22**
**2**	**P6 Suspiciousness**	**−0.82**	**0.21**
**3**	**P3 Hallucinations**	**−0.74**	**0.21**
**4**	**G2 Anxiety**	**−0.63**	**0.19**
**5**	**G4 Tension**	**−0.44**	**0.16**
**6**	**P4 Excitement**	**−0.33**	**0.14**
**7**	**P2 Conceptual disorganization**	**−0.33**	**0.16**
8	P7 Hostility	0.09	0.05
9	G9 Unusual thought content	0.16	0.09
10	G14 Poor impulse control	0.22	0.07
11	G15 Preoccupation	0.31	0.13
12	G16 Active social avoidance	0.34	0.09
13	G6 Depression	0.36	0.09
14	G8 Uncooperativeness	0.43	0.19
15	G12 Lack of judgment & insight	0.56	0.17
16	N7 Stereotyped thinking	0.58	0.18
17	G11 Poor attention	0.62	0.19
18	G13 Disturbance of volition	0.71	0.20
19	N6 Lack of spontaneity & flow of conversation	0.82	0.29
20	N3 Poor rapport	0.84	0.23
21	N1 Blunted affect	1.11	0.33
22	N2 Emotional withdrawal	1.14	0.36
23	G1 Somatic concern	1.19	0.40
24	N4 Passive-apathetic social withdrawal	1.23	0.41
25	P5 Grandiosity	1.48	0.55
26	G3 Guilt feelings	1.61	0.57
27	N5 Difficulty in abstract thinking	1.66	0.62
28	G5 Mannerisms & posturing	2.10	0.91
29	G10 Disorientation	2.58	1.15
30	G7 Motor retardation	3.23	1.91

## Discussion

The rapid growth in the use of mobile devices has opened a new world of opportunities in mental health management for diseases such as schizophrenia.^17^ In order to prioritize the symptoms to be captured by periodic self-report in technology-enabled remote assessment solutions for tracking symptoms and detecting relapse early in schizophrenia patients, we utilized existing data from three relapse-prevention clinical trials to understand which PANSS items changed the most immediately before relapse and when exactly these symptoms started to manifest.

Not all the individual PANSS items would have the same level of change prior to impending relapse. A subset of seven PANSS items had more increases than others in our data. These seven PANSS items were more in line with the early warning signs identified through a prospective study.^27^ This may be owing to the fact that our data were also collected prospectively. Five of the seven PANSS items, P1, P2, P3, P4, and P6, were from the positive symptoms subscale. This was as expected because relapse was typically expressed as exacerbation of the positive symptoms of schizophrenia. The remaining two items, G2 and G4, were among the early warning signs identified through both prospective and retrospective studies. Thus our data provided further confirmation for a major role of affectivity in psychotic relapse.

Relapse appeared to be more abrupt in our data than what have been reported previously. Previous reports suggested that symptoms of relapse start to develop in 2–4 weeks prior to relapse.^4,26,28,43^ It is worth noting that most of the previous studies reporting the time interval between the earliest symptom worsening and relapse are retrospective studies. A limitation of retrospective studies is that patients or relatives may well be able to name the early signs when looking back, but may not be able to recognize them when they actually occur.^30^ To provide an accurate estimate of the time interval between the earliest symptom worsening and relapse, we used prospectively collected data and applied a longitudinal approach to model the trajectories of individual PANSS items after carefully aligning the patient observations by their time of observation as days relative to relapse. In our data, the trajectories of the seven PANSS items that had the most changes prior to impending relapse started to increase ~7−10 days before relapse and reached on average 1-point of increase about 0.3 ~ 1.2 days before relapse (Fig. 2, Table 3). The time interval between the earliest symptom worsening and relapse that was estimated form our model based on prospectively collected data was much shorter than what was reported in the retrospective studies. Based on our results, the symptoms of schizophrenia patients need to be assessed at least weekly to be able to detect relapse early. This is beyond the capacity of current clinical practice. In this situation, mobile technology may be instrumental in tracking symptoms of schizophrenia patients as it can collect periodic self-reports at a higher frequency than what is feasible with current standard care. Several studies have shown good compliance rates in collecting self-reports on symptoms in schizophrenia patients.^23,44,45^ Combining the self-report data and the continuously collected passive sensing data, we have a unique opportunity to develop a remote assessment solution to monitor the symptoms and detect relapse early in schizophrenia patients.

There are a few limitations to the current study. The main limitation is that these results were derived in a post hoc manner from several pooled clinical trials and should be used as hypothesis generation only. Patients with co-morbid psychiatric diagnoses, significant medical diagnosis, active substance dependence, significant risk of suicidal/aggressive behavior, or recent involuntary hospitalization were excluded from these clinical trials. The population used in this study may not be exactly the same as a typical population that would be put through remote monitoring. In addition, the symptoms of schizophrenia were measured in these clinical trials by the PANSS, which was a “non-technology” instrument designed to be administered by physicians and to include information from informants. The PANSS items that changed the most prior to impending relapse may not be the same as the symptoms that changed the most based on patients’ self-report collected by mobile devices. Future studies that use mobile devices to collect self-reported symptoms in schizophrenia patients suitable for remote monitoring are needed to verify whether the same set of symptoms change the most prior to impending relapse. Second, random effects were included in the mixed effect model analysis to account for the correlations among repeated measurements. The linear and non-linear mixed effect models included two and one random effect, respectively. Mixed effect models with additional random effects did not converge with the data available. Thus, it is not clear whether the correlations among the repeated measures were fully accounted. As a result, the 95% confidence intervals of the trajectories may be underestimated. PANSS data collected in more relapsed patients and in higher frequency prior to relapse is needed to get more accurate estimates of the confidence intervals of the trajectories. Third, the seven PANSS items that had most changes before relapse were identified at the population level. Individual patients may have different relapse signatures. We are not able to investigate the individual relapse profile in current dataset because the patients were only followed until their first relapse in the three studies. Long-term follow-up data capturing multiple relapses of the same patients is needed to understand the individual profiles of relapse. At last, we propose to monitor a subset of symptoms by periodic self-report with the consideration that asking the patients to provide frequent self-reports on all schizophrenia symptoms would be too much a burden and thus infeasible. As a result, other potential markers of relapse, e.g., negative symptoms, would be missed by the periodic self-report. In practice, periodic self-report will be used together with continuous passive monitoring, which may capture some other potential markers of relapse such as insomnia, change in mobility, change in fluency of conversation, reduced social interactions, etc.

In summary, a subset of seven PANSS items exhibited greater increases than other items prior to impending relapse. These seven PANSS items included P1 (delusions), P2 (conceptual disorganization), P3 (Hallucinations), P4 (excitement), P6 (suspiciousness) from the positive symptoms subscale and G2 (anxiety) and G4 (tension) from the general psychopathology subscale. This subset of PANSS items could be used to develop remote assessment solutions for monitoring symptoms and detecting relapse early in schizophrenia patients. Further prospective studies are needed to assess the predictive validity of these items.

## Methods

### Data

Data were pooled from three randomized, double-blind, placebo-controlled withdrawal studies (NCT00086320, NCT00111189, and NCT01529515) to determine the efficacy of paliperidone oral ER formulation, paliperidone palmitate 1-month injectable formulation (PP1M), and paliperidone palmitate 3-month injectable formulation (PP3M), respectively, in delaying psychotic relapse in adult patients with a diagnosis of schizophrenia by DSM-IV-TR criteria for at least 1 year. The three studies had similar study designs. Each study had a screening phase, an open-label run-in or transition phase during which eligible patients were transitioned to the study drug (or PP1M instead of PP3M for the PP3M study) and had their symptoms controlled, an open-label stabilization or maintenance phase during which stable patients received flexible doses of the study drug, and a double-blind phase during which stabilized patients were randomized in a 1:1 ratio to receive either study drug or placebo and were followed until they experienced a relapse, they withdrew from the study, or the study was completed. The differences in the study designs among the three studies were summarized in Supplementary Table 1. The detailed findings from these three studies were reported previously.^46–48^ All the three formulations of paliperidone significantly delayed time-to-relapse of psychotic symptoms compared to placebo. These three studies were included in current analysis because: (1) PANSS was assessed relatively frequently (at least every 4 weeks) so that the last pre-relapse visit was not too far away from the time of relapse; (2) patients who entered the double-blind phase had been stable for at least 8 weeks owing to the stabilization/maintenance phase built in the study designs, making this population similar to a patient population that would be put through remote monitoring and helping to get a clearer picture on when exactly the symptoms started to increase prior to relapse. All studies were conducted in accordance with the ethical principles in the Declaration of Helsinki, consistent Good Clinical Practices and applicable regulatory requirements. The study protocols and amendments were approved by either an independent ethics committee or an institutional review board for each site. All participants provided written informed consent.

### PANSS

PANSS^25^ is widely used in the study of antipsychotic therapy for measuring symptom severity of patients with schizophrenia. Patients are rated 1–7 on 30 symptoms based on a clinical interview as well as reports of family members or primary care hospital workers. The 30 items are grouped into three subscales: positive scale (seven items), negative scale (seven items), and general psychopathology scale (16 items). The PANSS total score ranges from 30 to 210. The PANSS was administered every 4 weeks in the three studies except in the paliperidone oral ER formulation study it was administered weekly or biweekly into week 8 of the double-blind phase and every 4 weeks thereafter.

### Definition of relapse

The primary efficacy variable of these three studies was the time-to-first-relapse during the double-blind phase. Relapse was defined by any one of the following criteria: (1) psychiatric hospitalization (involuntary or voluntary admission to a psychiatric hospital for decomposition of the subject’s schizophrenia symptoms); (2) deliberate self-injury or aggressive behavior, or suicidal or homicidal ideation and aggressive behavior that was clinically significant; (3) 25% increase in PANSS total score for two consecutive assessments < 7 days apart for patients who scored > 40 at randomization, or a 10-point increase for patients who scored ≤ 40 at randomization; (4) increase for two consecutive assessments < 7 days apart in pre-specified individual PANSS item scores (P1 (Delusions), P2 (Conceptual disorganization), P3 (Hallucinations), P6 (Suspiciousness), P7 (Hostility), and G8 (Uncooperativeness)) to ≥ 5 for patients whose score was ≤ 3 at randomization, or to ≥ 6 for patients whose score was four at randomization. In the paliperidone oral ER study, relapse was also defined by a significant increase in the clinical global impression-severity (CGI-S) score. To make the relapse definition more homogenous across the three studies, this criterion was dropped for the paliperidone oral ER study. As a result, one patient’s status was changed from relapse to non-relapse and was excluded from current analysis. There were 10 patients who did not meet the above criteria for relapse but were classified as patients who experienced a relapse. These 10 patients were excluded from further analysis. There were also 14 patients who met the above criteria for relapse but were not called as relapse during the study. These 14 patients were reclassified as patients who experienced a relapse.

### Statistical analysis

Individual PANSS items were sorted by their changes at relapse from randomization (a) in patients who experienced a relapse during the double-blind phase of the three studies to see which PANSS items had the most increases at the time of relapse; (b) in patients with different relapse reasons separately to see whether the same set of PANSS items had the most increases at relapse in patients with different relapse reasons, especially in patients whose relapse was not defined by a significant increase in at least one of the pre-specified PANSS items; (c) in relapsed patients receiving paliperidone and those receiving placebo separately to see the whether the PANSS items with the most increases at relapse differed between patients receiving different treatments. The changes in individual PANSS items from the last pre-relapse visit to relapse and from randomization to the last pre-relapse visit were compared to see in which period the majority of the increases occurred.

Linear and non-linear mixed effect models were applied to model the trajectories of individual PANSS items from a stable state to the time of relapse in patients who experienced a relapse during the double-blind phase of the three studies. As relapse occurred at different timepoints for different patients, the patient observations were aligned by their time of observation as days relative to relapse instead of their scheduled visits. Time (days relative to relapse) was modeled as a continuous variable in the mixed effect models. PANSS item scores at the time of relapse, at the last pre-relapse visit, and during up to 8 weeks before the last pre-relapse visit were included in the analysis. For the paliperidone oral ER study, up to seven PANSS assessments of each patient were included in the analysis. For the remaining two studies, up to four PANSS assessments of each patient were included in the analysis. The earliest PANSS assessment included in the analysis was at week 6 of the run-in phase for the oral ER study, at week 8 of the maintenance phase for the PP1M study, and at week 17 of the transition phase of the PP3M study, respectively. Patients who entered the double-blind phase later already reached stable state at these visits. Let *Y*_*ij*_ be the *j*th observation of a PANSS item on the *i*th patient and *T*_*ij*_ be the days relative to relapse for the observation with *i*=1,…*n* and *j*=1,…*n*_*i*_. In the linear mixed effect model, the trajectory of an individual PANSS item was modeled as

$$Y_{ij} \sim polynomial\left( {T_{ij}} \right) + \beta _{0,i} + \beta _{1,i} \ast T_{ij} + \varepsilon _{ij},$$

where *polynomial*(*T*_*ij*_) was a polynomial function of *T*_*ij*_ with an order up to 7, $\beta _{0,i} \sim N(0,\sigma _0^2)$ and $\beta _{1,i} \sim N(0,\sigma _1^2)$ were subject-level intercept and slope for modeling the correlations among repeated measures, and *ε*_*ij*_ was the observational error. The order of the polynomial function was determined through model selection using the Akaike information criteria.^49^ In the non-linear mixed effect model, the trajectory of an individual PANSS item was modeled as an exponential function:

$$Y_{ij} \sim a^{(T_{ij} - b)} + c + \delta _i + \varepsilon _{ij},$$

where $\delta _i \sim N(0,\sigma _\delta ^2)$ was a subject-level random effect included in the model to account for the correlations among repeated measures and *ε*_*ij*_ was the observational error. Compared with the polynomial function used in the linear mixed effect model, the exponential function used in the non-linear mixed effect model made stronger assumption regarding the shape of the trajectory, i.e., the individual PANSS item scores increased exponentially before relapse. However, the parameters of the exponential function could be easily interpreted. The parameter *a* was an indicator of the speed of PANSS item increase before relapse. Given the same magnitude of increase at the time of relapse, a smaller *a* parameter indicated the PANSS item increased relatively slowly before relapse thus may start to increase early. The parameter *b* represented the number of days before relapse when the PANSS item had 1-point of increase from its pre-relapse level. A negative *b* parameter indicated the patients would have on average > 1-point of increase on the PANSS item before relapse. The parameter *c* was the average pre-relapse level of the PANSS item.

All statistical analyses were performed using SAS 9.4 (www.sas.com).

### Data availability

The data that support the findings of this study are available from the Yale University Open Data Access (YODA) Project (http://yoda.yale.edu) with the identifiers NCT00086320, NCT00111189, and NCT01529515.

## Electronic supplementary material

Supplementary Materials
