# Supplementary material for: Trajectories and changes in individual items of positive and negative syndrome scale among schizophrenia patients prior to impending relapse
Source: NPJ Schizophr. 2018 Jun 20;4:10. doi: 10.1038/s41537-018-0056-6 (PMC6010453; doi:10.1038/s41537-018-0056-6)
Supplement: Supplementary file 1 — Supplementary Materials [file 41537_2018_56_MOESM1_ESM.pdf]

SUPPLEMENTARY MATERIALS

Trajectories and Changes in Individual Items of Positive and Negative Syndrome Scale among Schizophrenia Patients Prior to Impending Relapse

Running Title: PANSS Item Trajectories in Schizophrenia Relapse

Dai Wang[1], Srihari Gopal[2], Susan Baker[2], Vaibhav A. Narayan[1]

[1]R&D Information Technology and [2]Neuroscience Therapeutic Area, Janssen Research & Development, LLC, Titusville, NJ

Address correspondence to:
Dai Wang, Ph.D.
R&D Information Technology
Janssen Research & Development, LLC
1000 US Route 202, C-224
Raritan, NJ 08869
Tel: (908) 218-7462
dwang39@its.jnj.com

Supplementary Table 1. Differences in study designs among the 3 studies

	NCT00086320	NCT00111189	NCT01529515
Compound	paliperidone oral extended-release (ER) formulation	paliperidone palmitate 1-month injectable formulation (PP1M)	paliperidone palmitate 3-month injectable formulation (PP3M)
Study phases	8 week run-in;<br>6 week stabilization;<br>Double-blind relapse prevention	9 week transition;<br>24 week maintenance;<br>Double-blind relapse prevention	17 week transition (treated with PP1M instead of PP3M);<br>12 week maintenance;<br>Double-blind relapse prevention
Inclusion criteria	PANSS total score in (70, 120)	PANSS total score <120	PANSS total score <120
Criteria to enter stabilization /maintenance phase	* PANSS total score <70 for at least 2 weeks;<br>* A score of ≤4 for at least 2 weeks for PANSS items P1, P2, P3, P6, P7, G8;<br>* CGI-S ≤4 for at least 2 weeks;<br>* The dose of paliperidone ER remains unchanged for at least 2 weeks	PANSS total score ≤75 at week 9	PANSS total score ≤70 at week 17
Criteria to enter double-blind phase	* PANSS total score ≤70;<br>* A score of ≤4 for each of the PANSS items P1, P2, P3, P6, P7, G8;<br>* CGI-S ≤4;<br>* Stable dose of paliperidone ER	* PANSS total score ≤75;<br>* A score of ≤4 for each of the PANSS items P1, P2, P3, P6, P7, G8, G14;<br>* Stable dose of PP1M	* PANSS total score ≤70;<br>* A score of ≤4 for each of the PANSS items P1, P2, P3, P6, P7, G8, G14

PANSS – Positive and Negative Syndrome Scale, P1 – Delusions, P2 – Conceptual disorganization, P3 – Hallucinations, P6 – Suspiciousness, P7 – Excitement, G8 – Uncooperativeness, G14 – Poor impulse control, CGI – Clinical Global Impression, CGI-S – Clinical Global Index - Severity.

Supplementary Table 2. Changes in individual PANSS items at relapse from randomization in patients with different relapse reasons

PANSS item	All relapse reasons (N=255)		Relapse defined by hospitalization, suicidal /homicidal ideation, aggressive behavior (N=51)		Relapse defined by a significant increase in one of the pre-specified PANSS items (N=65)		Relapse defined by a significant increase in the PANSS total score but not in any of the pre-specified PANSS items (N=139)	
	Rank	Mean change (standard error)	Rank	Mean change (standard error)	Rank	Mean change (standard error)	Rank	Mean change (standard error)
**P1 Delusions**	**1**	**1.53 (0.08)**	**1**	**1.80 (0.19)**	**1**	**1.92 (0.16)**	**3**	**1.25 (0.09)**
**P6 Suspiciousness**	**2**	**1.49 (0.08)**	**6**	**1.49 (0.19)**	**3**	**1.69 (0.18)**	**1**	**1.40 (0.09)**
**P3 Hallucinations**	**3**	**1.44 (0.09)**	**2**	**1.76 (0.19)**	**2**	**1.88 (0.20)**	**6**	**1.11 (0.10)**
**G2 Anxiety**	**4**	**1.32 (0.07)**	**5**	**1.51 (0.18)**	**8**	**1.12 (0.15)**	**2**	**1.34 (0.09)**
**P4 Excitement**	**5**	**1.29 (0.07)**	**3**	**1.65 (0.18)**	**5**	**1.29 (0.16)**	**5**	**1.16 (0.08)**
**G4 Tension**	**6**	**1.24 (0.07)**	**7**	**1.39 (0.16)**	**6**	**1.20 (0.15)**	**4**	**1.20 (0.09)**
**P2 Conceptual disorganization**	**7**	**1.12 (0.07)**	**10**	**1.29 (0.14)**	**4**	**1.49 (0.16)**	**7**	**0.88 (0.08)**
G9 Unusual thought content	8	1.00 (0.07)	8	1.35 (0.17)	11	0.98 (0.13)	8	0.88 (0.08)
P7 Hostility	9	0.99 (0.07)	4	1.53 (0.17)	10	0.98 (0.17)	12	0.79 (0.08)
G15 Preoccupation	10	0.95 (0.06)	15	0.98 (0.14)	7	1.17 (0.14)	9	0.84 (0.08)
G14 Poor impulse control	11	0.92 (0.07)	9	1.33 (0.19)	17	0.88 (0.14)	13	0.78 (0.09)
G8 Uncooperativeness	12	0.88 (0.07)	12	1.08 (0.17)	9	1.08 (0.15)	16	0.71 (0.08)
G11 Poor attention	13	0.85 (0.06)	17	0.84 (0.13)	12	0.98 (0.14)	11	0.80 (0.08)
G12 Lack of judgment & insight	14	0.85 (0.06)	11	1.10 (0.13)	16	0.88 (0.14)	14	0.75 (0.08)
N7 Stereotyped thinking	15	0.83 (0.06)	13	1.04 (0.12)	20	0.69 (0.15)	10	0.81 (0.08)
G16 Active social avoidance	16	0.82 (0.06)	14	1.00 (0.14)	13	0.94 (0.13)	17	0.71 (0.08)
N3 Poor rapport	17	0.75 (0.06)	19	0.75 (0.11)	14	0.89 (0.11)	18	0.69 (0.08)
G6 Depression	18	0.73 (0.07)	16	0.90 (0.18)	22	0.63 (0.15)	15	0.72 (0.09)
N6 Lack of spontaneity & flow of conversation	19	0.69 (0.06)	18	0.78 (0.13)	15	0.89 (0.14)	21	0.57 (0.09)
G13 Disturbance of volition	20	0.64 (0.06)	23	0.61 (0.13)	18	0.78 (0.14)	19	0.58 (0.09)
N4 Passive-apathetic social withdrawal	21	0.59 (0.07)	21	0.67 (0.15)	19	0.69 (0.15)	22	0.52 (0.08)
N5 Difficulty in abstract thinking	22	0.54 (0.05)	22	0.67 (0.13)	25	0.51 (0.11)	23	0.50 (0.07)
G1 Somatic concern	23	0.53 (0.07)	26	0.51 (0.20)	26	0.46 (0.13)	20	0.58 (0.08)
P5 Grandiosity	24	0.49 (0.06)	24	0.57 (0.15)	21	0.65 (0.14)	24	0.40 (0.08)
N2 Emotional withdrawal	25	0.44 (0.06)	25	0.53 (0.15)	24	0.51 (0.14)	25	0.38 (0.08)
G3 Guilt feelings	26	0.41 (0.06)	20	0.69 (0.18)	30	0.26 (0.11)	26	0.37 (0.08)
G5 Mannerisms & posturing	27	0.32 (0.05)	27	0.47 (0.12)	27	0.40 (0.11)	28	0.23 (0.07)
N1 Blunted affect	28	0.31 (0.06)	30	0.22 (0.14)	28	0.34 (0.14)	27	0.32 (0.07)
G10 Disorientation	29	0.28 (0.05)	29	0.27 (0.09)	23	0.52 (0.12)	30	0.17 (0.06)
G7 Motor retardation	30	0.24 (0.06)	28	0.39 (0.15)	29	0.28 (0.10)	29	0.17 (0.08)

Supplementary Table 3. Changes in individual PANSS items at relapse from randomization in patients receiving oral ER or injectable (PP1M/PP3M) formulations of paliperidone**.** The PANSS items are ordered by the rank of mean change in all relapsed patients

		Patients receiving paliperidone oral ER (N=26)		Patients receiving paliperidone injectable (N=43)	
Order	PANSS item	Rank	Mean change (standard error)	Rank	Mean change (standard error)
**1**	**P1 Delusions**	**3**	**1.31 (0.21)**	**1**	**1.37 (0.21)**
**2**	**P6 Suspiciousness**	**4**	**1.31 (0.21)**	**2**	**1.28 (0.21)**
**3**	**P3 Hallucinations**	**1**	**1.50 (0.32)**	**5**	**1.12 (0.20)**
**4**	**G2 Anxiety**	**5**	**1.23 (0.22)**	**3**	**1.28 (0.19)**
**5**	**P4 Excitement**	**2**	**1.35 (0.20)**	**9**	**0.91 (0.18)**
**6**	**G4 Tension**	**12**	**0.96 (0.20)**	**6**	**1.12 (0.17)**
**7**	**P2 Conceptual disorganization**	**6**	**1.19 (0.25)**	**7**	**1.02 (0.16)**
8	G9 Unusual thought content	20	0.65 (0.17)	12	0.74 (0.18)
9	P7 Hostility	13	0.88 (0.21)	17	0.63 (0.14)
10	G15 Preoccupation	22	0.62 (0.17)	4	1.14 (0.18)
11	G14 Poor impulse control	19	0.69 (0.18)	8	0.98 (0.21)
12	G8 Uncooperativeness	17	0.77 (0.22)	14	0.72 (0.18)
13	G11 Poor attention	18	0.69 (0.17)	20	0.63 (0.18)
14	G12 Lack of judgment & insight	15	0.81 (0.21)	16	0.65 (0.15)
15	N7 Stereotyped thinking	7	1.08 (0.19)	15	0.65 (0.17)
16	G16 Active social avoidance	10	1.00 (0.20)	13	0.74 (0.14)
17	N3 Poor rapport	8	1.00 (0.20)	11	0.77 (0.17)
18	G6 Depression	14	0.85 (0.25)	10	0.84 (0.20)
19	N6 Lack of spontaneity & flow of conversation	11	0.96 (0.22)	21	0.58 (0.15)
20	G13 Disturbance of volition	23	0.54 (0.19)	23	0.53 (0.16)
21	N4 Passive-apathetic social withdrawal	9	1.00 (0.22)	19	0.63 (0.17)
22	N5 Difficulty in abstract thinking	25	0.38 (0.22)	25	0.44 (0.15)
23	G1 Somatic concern	16	0.77 (0.22)	26	0.30 (0.16)
24	P5 Grandiosity	28	0.31 (0.17)	24	0.44 (0.16)
25	N2 Emotional withdrawal	21	0.62 (0.21)	18	0.63 (0.15)
26	G3 Guilt feelings	26	0.35 (0.15)	29	0.21 (0.13)
27	G5 Mannerisms & posturing	30	-.04 (0.09)	27	0.28 (0.15)
28	N1 Blunted affect	24	0.50 (0.18)	22	0.56 (0.14)
29	G10 Disorientation	29	0.12 (0.06)	30	0.14 (0.14)
30	G7 Motor retardation	27	0.35 (0.16)	28	0.28 (0.15)

Supplementary Table 4. Changes in individual PANSS items in relapsed patients from the last pre-relapse visit to relapse and from randomization to the last pre-relapse visit. The PANSS items are ordered by the rank of mean change from randomization to relapse

Order	PANSS item	From last pre-relapse visit to relapse (N=255)		From randomization to last pre-relapse visit (N=267)	
		Rank	Mean change (standard error)	Rank	Mean change (standard error)
**1**	**P1 Delusions**	**1**	**1.35 (0.08)**	**3**	**0.17 (0.04)**
**2**	**P6 Suspiciousness**	**2**	**1.28 (0.07)**	**1**	**0.19 (0.05)**
**3**	**P3 Hallucinations**	**3**	**1.25 (0.08)**	**2**	**0.18 (0.04)**
**4**	**G2 Anxiety**	**4**	**1.18 (0.07)**	**5**	**0.14 (0.04)**
**5**	**P4 Excitement**	**6**	**1.13 (0.07)**	**4**	**0.16 (0.05)**
**6**	**G4 Tension**	**5**	**1.13 (0.07)**	**6**	**0.11 (0.04)**
**7**	**P2 Conceptual disorganization**	**7**	**1.01 (0.07)**	**7**	**0.09 (0.04)**
8	G9 Unusual thought content	9	0.88 (0.06)	8	0.09 (0.04)
9	P7 Hostility	8	0.91 (0.07)	9	0.08 (0.03)
10	G15 Preoccupation	10	0.87 (0.06)	12	0.07 (0.04)
11	G14 Poor impulse control	11	0.85 (0.07)	11	0.07 (0.04)
12	G8 Uncooperativeness	12	0.82 (0.07)	13	0.07 (0.03)
13	G11 Poor attention	15	0.79 (0.06)	16	0.04 (0.04)
14	G12 Lack of judgment & insight	13	0.82 (0.06)	18	0.04 (0.04)
15	N7 Stereotyped thinking	14	0.81 (0.06)	17	0.04 (0.03)
16	G16 Active social avoidance	17	0.76 (0.06)	14	0.06 (0.04)
17	N3 Poor rapport	18	0.71 (0.05)	15	0.05 (0.04)
18	G6 Depression	16	0.77 (0.07)	30	-0.05 (0.04)
19	N6 Lack of spontaneity & flow of conversation	19	0.70 (0.06)	23	0.00 (0.04)
20	G13 Disturbance of volition	20	0.68 (0.06)	29	-0.04 (0.04)
21	N4 Passive-apathetic social withdrawal	21	0.59 (0.06)	21	0.01 (0.04)
22	N5 Difficulty in abstract thinking	23	0.47 (0.05)	10	0.08 (0.04)
23	G1 Somatic concern	22	0.54 (0.06)	22	0.01 (0.05)
24	P5 Grandiosity	24	0.46 (0.06)	19	0.03 (0.04)
25	N2 Emotional withdrawal	25	0.45 (0.06)	20	0.01 (0.04)
26	G3 Guilt feelings	26	0.44 (0.06)	26	-0.04 (0.03)
27	G5 Mannerisms & posturing	27	0.36 (0.05)	27	-0.04 (0.04)
28	N1 Blunted affect	28	0.34 (0.06)	28	-0.04 (0.04)
29	G10 Disorientation	29	0.30 (0.04)	24	-0.02 (0.03)
30	G7 Motor retardation	30	0.27 (0.05)	25	-0.03 (0.03)

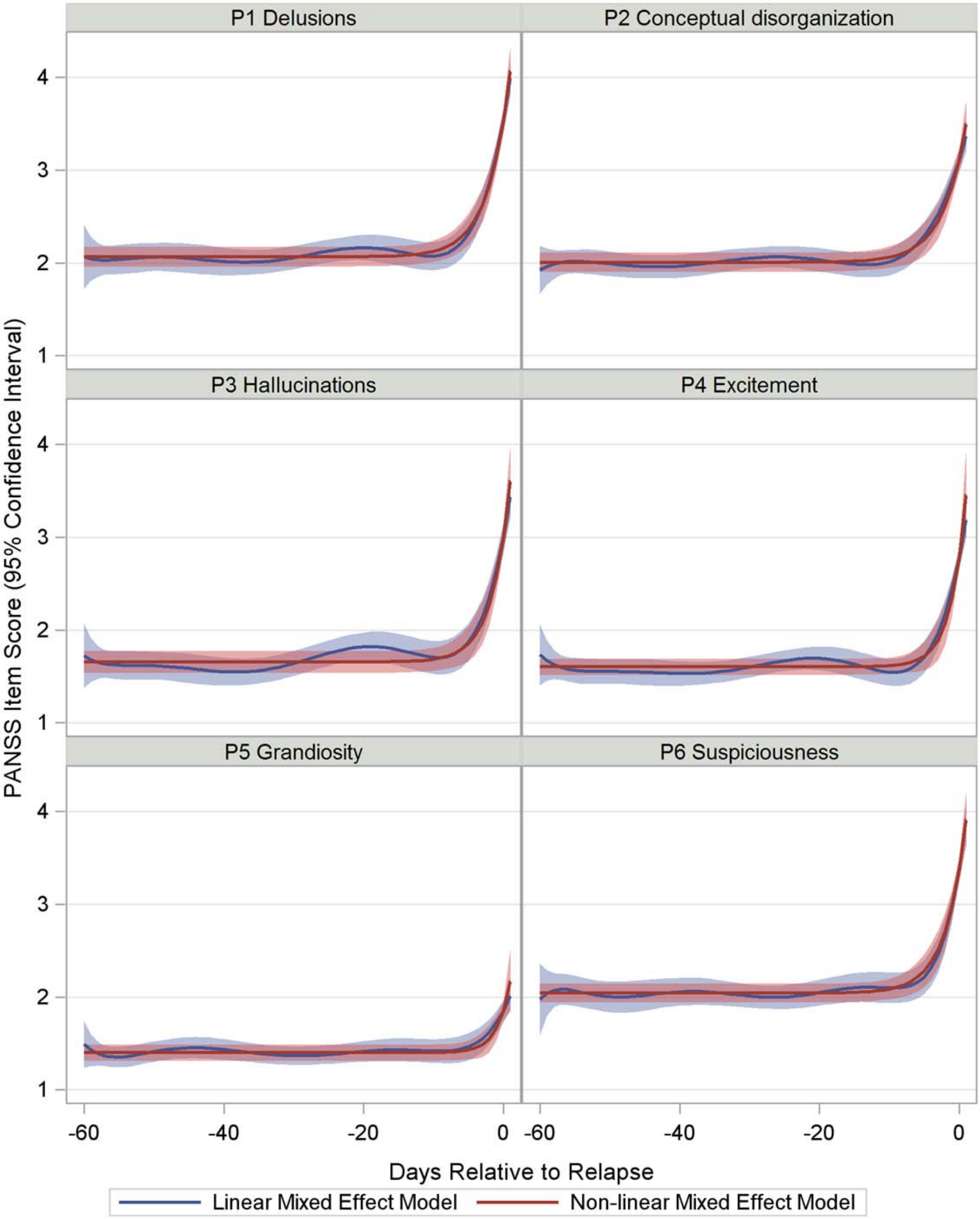

Supplementary Figure 1. Trajectories of PANSS items estimated from linear and non-linear mixed effect models

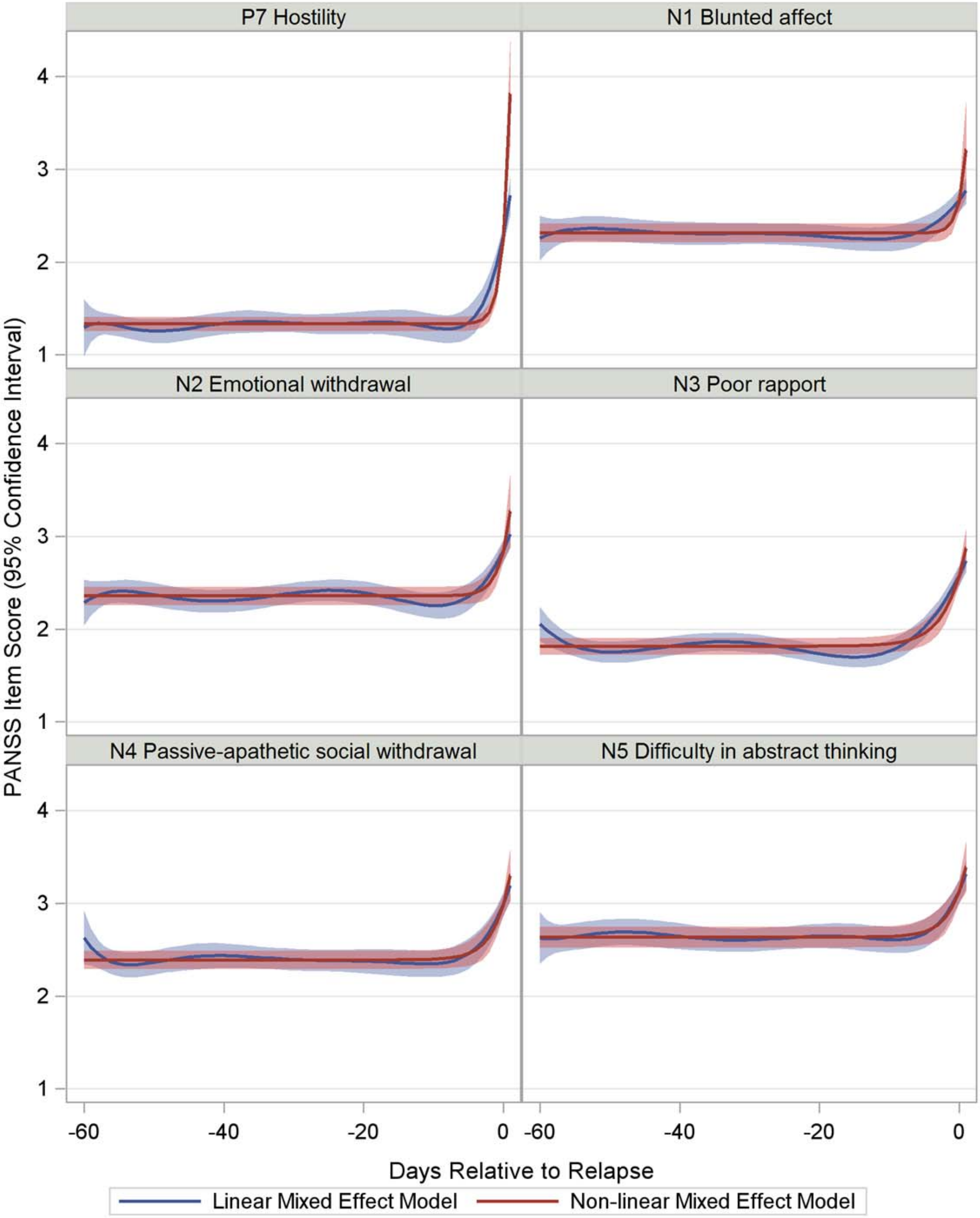

Supplementary Figure 1. Trajectories of PANSS items estimated from linear and non-linear mixed effect models (continued)

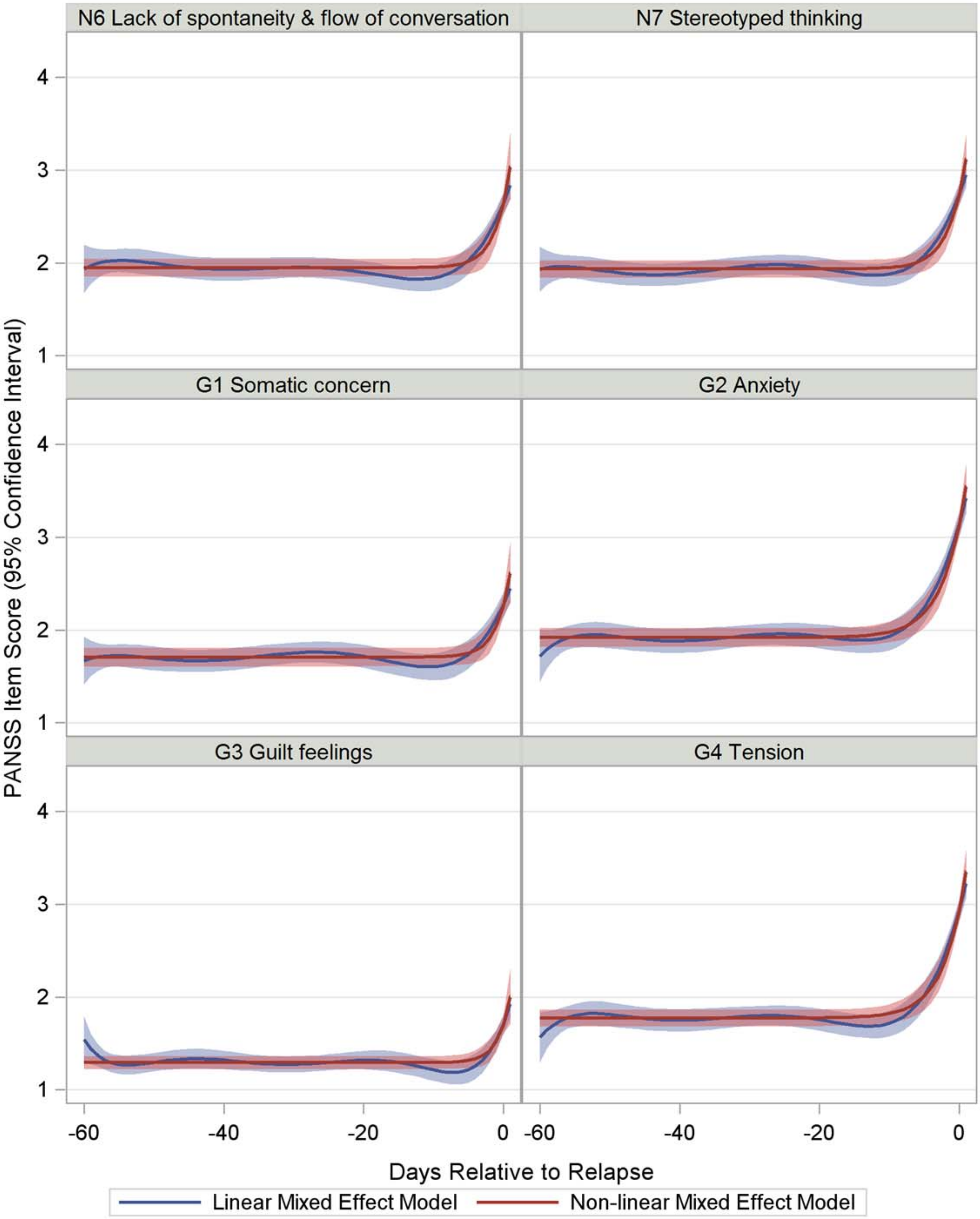

Supplementary Figure 1. Trajectories of PANSS items estimated from linear and non-linear mixed effect models (continued)

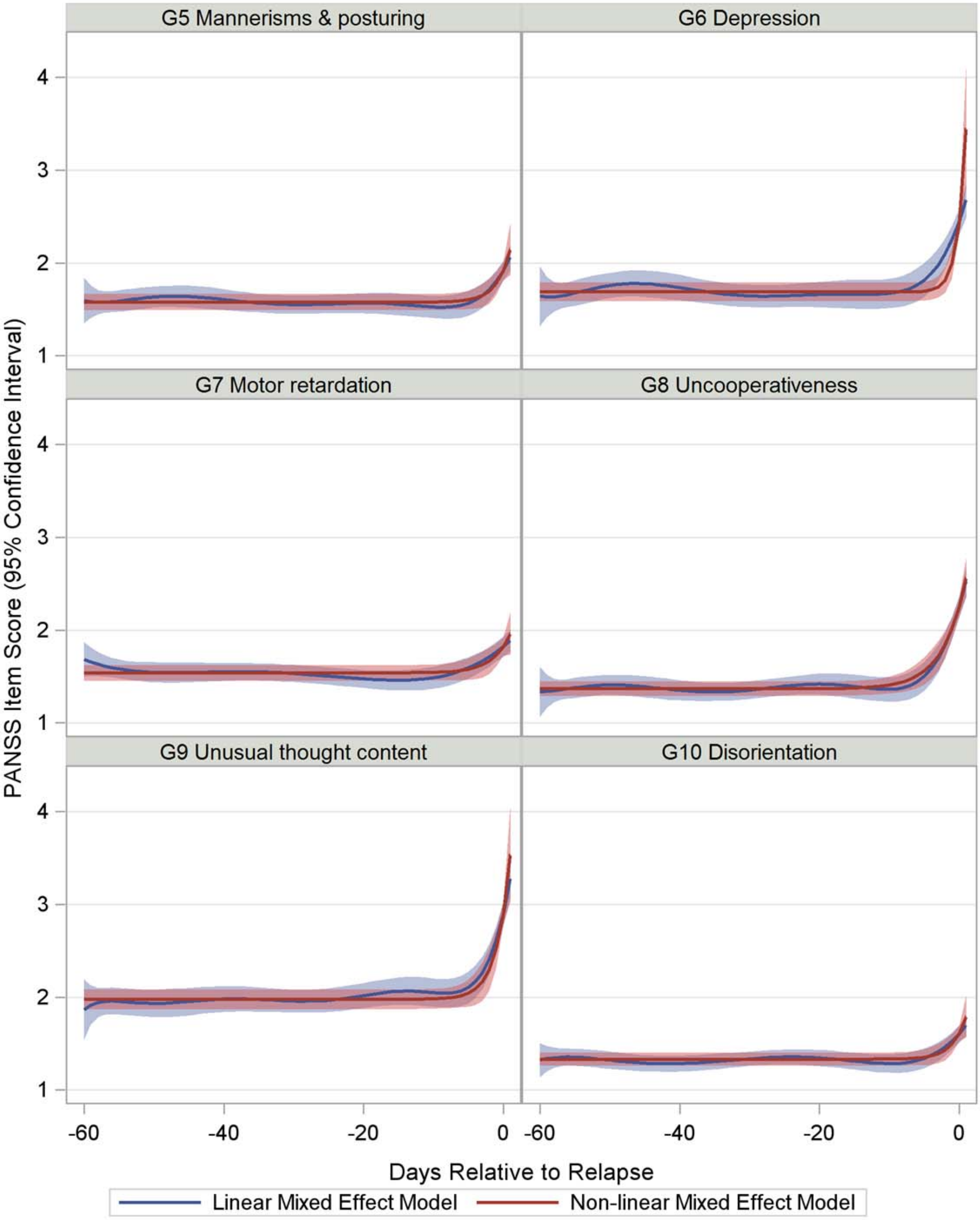

Supplementary Figure 1. Trajectories of PANSS items estimated from linear and non-linear mixed effect models (continued)

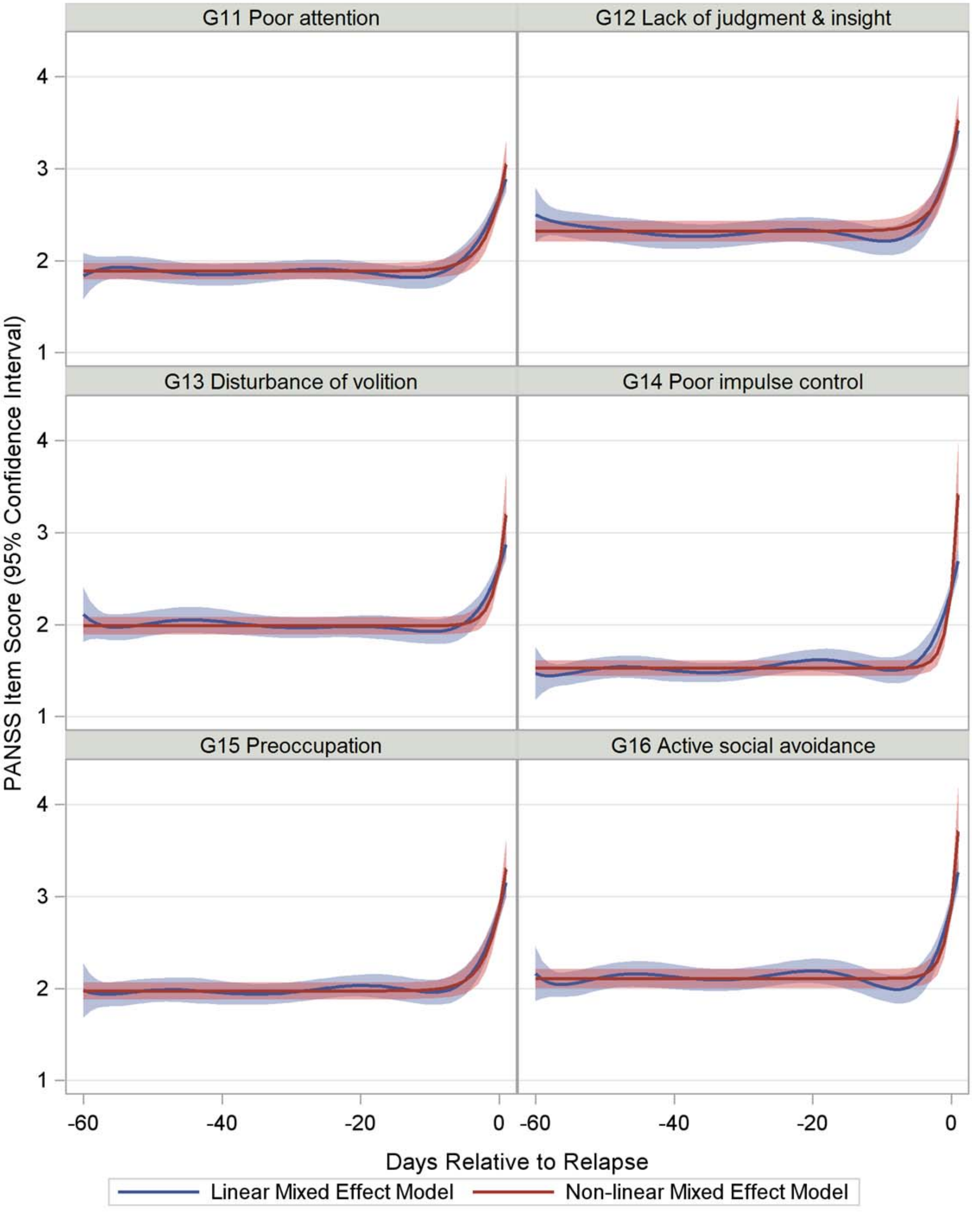

Supplementary Figure 1. Trajectories of PANSS items estimated from linear and non-linear mixed effect models (continued)

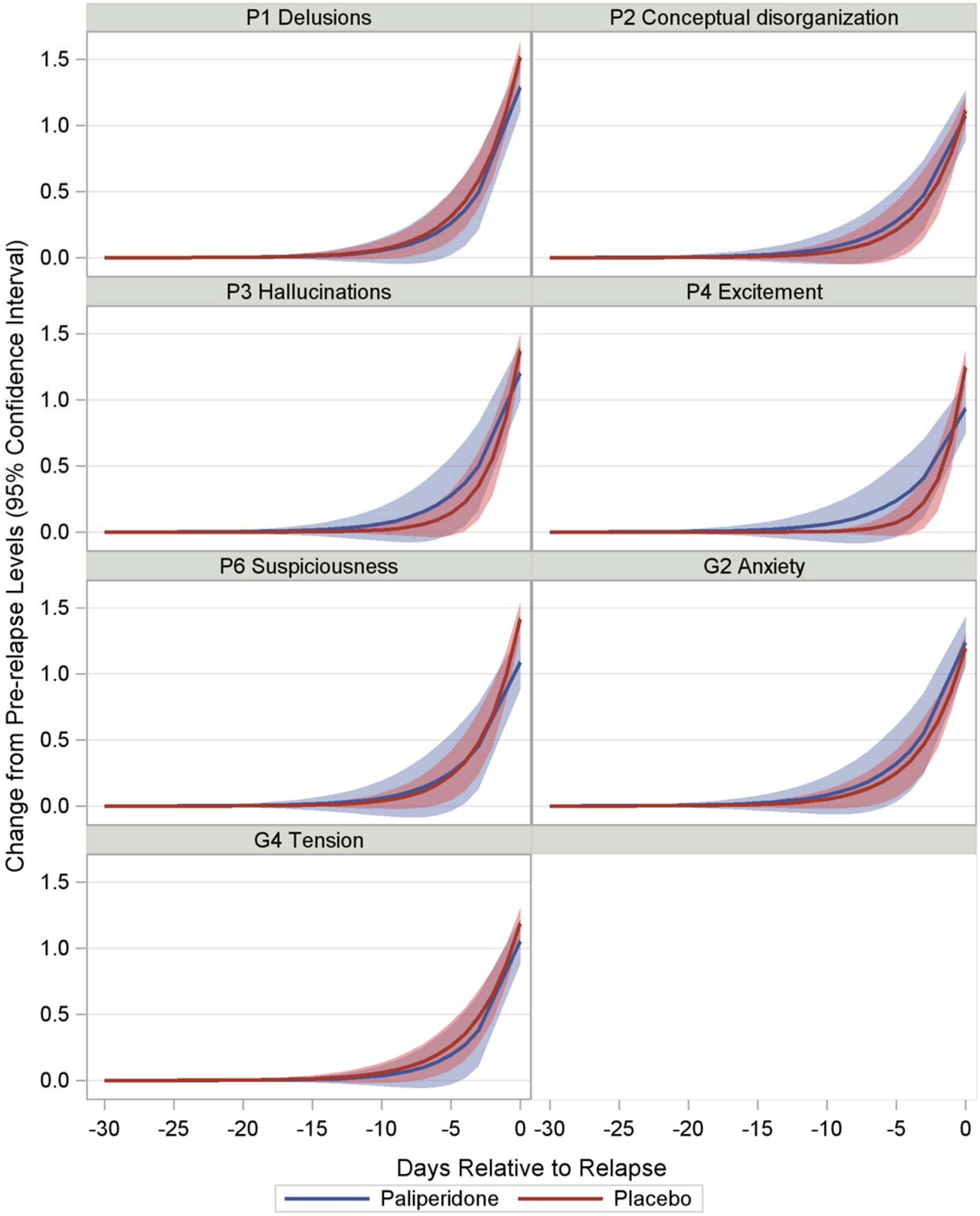

Supplementary Figure 2. Trajectories of changes from pre-relapse levels of the top 7 PANSS items that had most increases at relapse in patients receiving paliperidone (oral ER/PP1M/PP3M) and those receiving placebo. Trajectories were estimated from non-linear mixed effect models
